# Diversity-Orientated Synthesis and Biological Properties of Compounds Based on the *N*-Phenylquinoneimine Scaffold

**DOI:** 10.3390/molecules29010249

**Published:** 2024-01-03

**Authors:** Adebimpe Adesina, Rachid Skouta

**Affiliations:** 1 Department of Chemistry, Federal University of Agriculture, Abeokuta 111101, Nigeria; adesinaad@funaab.edu.ng; 2 Department of Biology, University of Massachusetts, Amherst, MA 01003, USA; 3 Department of Chemistry, University of Massachusetts, Amherst, MA 01003, USA

**Keywords:** *N*-phenylquinoneimine, diversity-orientated synthesis, biological activity, DNA, reactive metabolites, drugs

## Abstract

The *N*-phenylquinoneimine scaffold is a versatile synthetic platform that has gained significant attention in the field of drug discovery due to its structural diversity and capacity to interact with biologically relevant targets. This review explores established synthetic methodologies and highlights the significant biological activities exhibited by compounds derived from this scaffold, their implications for medicinal chemistry, and the development of novel therapeutics.

## 1. Introduction

Diversity-orientated synthesis (DOS) continues to grow as an area of importance in the disciplines of organic synthesis and chemical biology [1,2,3]. One important area that should benefit significantly from DOS is drug discovery. The existing chemical space can be expanded with new synthetic molecules, with the hope of identifying novel and better drug and probe molecules [4]. Arguably, one of the most promising synthetic strategies for generating collections of new compounds with increased molecular complexity and diversity via DOS involves the sequencing of multicomponent reactions with subsequent transformations, including cyclisations, couplings, and refunctionalisations [5].

DOS requires a planning algorithm to deliver an efficient but divergent route. Although DOS aims to achieve a diverse and non-focused coverage of biologically active chemical space, the results of DOS may find use in other fields in future years. Complexity-generating reactions are again important for efficiency (multicomponent-coupling, cascade and tandem complexity-generating reactions are the most valuable); however, pathways need to be identified that give structurally diverse targets. In order to achieve the highest levels of structural diversity, (i) the building blocks, (ii) the stereochemistry, (iii) the functional groups and, most importantly, (iv) the molecular framework must be varied. The key to the structural complexity is the complexity-generating reactions, while the key to the structural diversity comprises the branch points and building blocks [3]. The identification in the forward direction of pairwise relationships, where the product of one complexity-generating reaction is the substrate for another, can lead to high levels of molecular complexity in a very efficient manner [2].

### 1.1. N-Phenylquinoneimine and Its Pharmacological Significance

**Figure 1:**
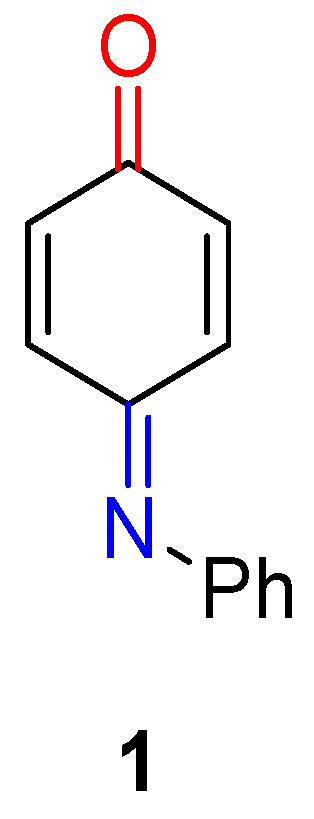
Structure of *N*-phenylquinoneimine **1** (NPQ).

*N*-Phenylquinoneimine (NPQ **1**, Figure 1), due to its α,β-keto and α,β-imino functionalities, is highly reactive and offers great potential for regioselective reactions. NPQs are highly coloured compounds [6,7,8] and constitute a core structure in several important natural products, Refs. [9,10], some of which are key abiotic and biological compounds, which intercalate with DNA [11]. New avenues for molecular sensors [12] and ligands, Refs. [13,14] for drug delivery [15,16] and controlled material growth have been provided through many of these hybrid materials. NPQs can be used as building blocks to access useful synthetic compounds that can serve as potential key intermediates in cascade reactions [17]. Since many natural products and druglike compounds include heterocyclic subunits, the ability to synthesise efficiently diverse heterocyclic compounds is critical. NPQs can be used to access aromatic heterocyclic structures largely used as scaffolds for generating combinatorial libraries in drug-discovery research [18]. Sulfones that can be synthesised from multistep reactions of NPQs are found in many medicines and drug candidates under development for the treatment of a host of diseases impacting human health worldwide [19]. NPQs represent a new frontier for the design and generation of molecular diversity and complexity [20,21].

### 1.2. Quinoneimines in Natural Products and Dyes

Natural products and their structural analogues have historically made a major contribution to pharmacotherapy, especially for cancer and infectious diseases [22,23], but also in other therapeutic areas, including cardiovascular diseases and multiple sclerosis [24,25,26]. Natural products are characterised by enormous scaffold diversity and structural complexity [27].

**Figure 2:**
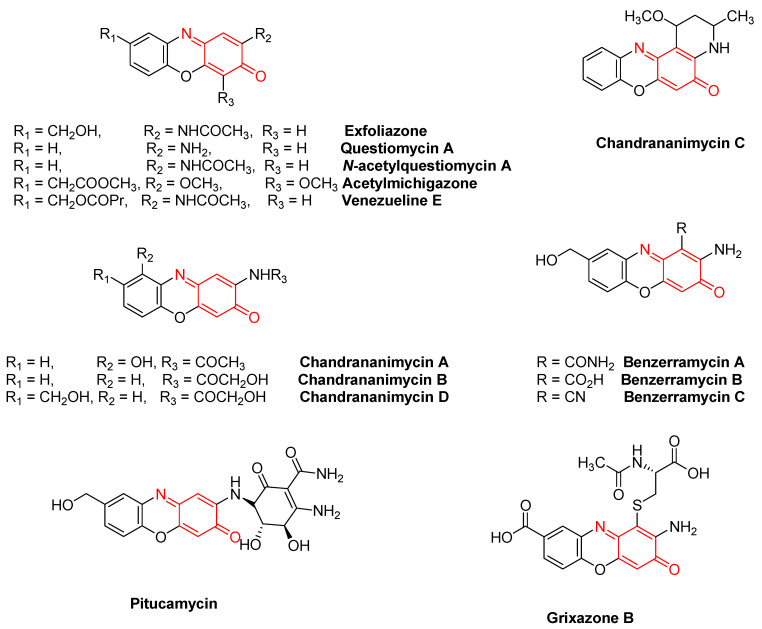
Some of the representative examples of quinoneimines in natural products.

Quinoneimines are highly coloured dyes [6,7,8,28,29] and constitute a core structure in several important natural products [9,10,30,31,32,33,34]. Some of these quinoneimines have been reported as growth-promoting substances with a low molecular weight, isolated from microorganisms [20]. Exfoliazone, Questiomycin A, *N*-Acetylquestiomycin A, and Acetylmichigazone have been isolated from *Streptomyces exfoliates* BT-38 [9,10], and Venezuelines A–G from Streptomyces venezuelae [30]. Chandrananimycins A–C were isolated from the culture broth of a marine *Actinomadura* sp. Isolate M045. They contain the phenoxazin-3-one chromophore, which is part of complex natural products like actinomycin, aurantin, and cryptomycin and is responsible for their colour [31]. Chandrananimycin D, pitucamycin, grixazone B, and benzerramycin A–C have been reported from a *Streptomyces griseus* strain isolated from an old building with moisture damage [32,33]. Cinnabarin and Cinnabarinic acid have been isolated as fungal pigments [34] (Figure 2).

**Figure 3:**
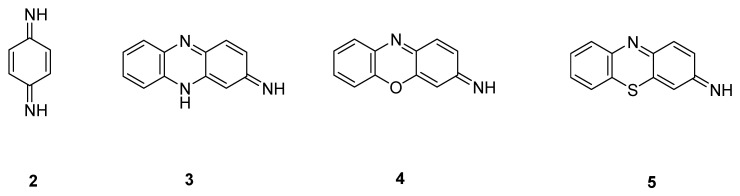
Some subgroups of quinoneimine dyes.

Quinoneimine dyes are based on the structure of the fictional compound para-quinone-di-imine **2**, from which the name of the dye class originates. There are several subgroups of quinoneimine dyes, such as the azines **3**, the oxazines **4**, and the thiazines **5** (Figure 3). 

Quinoneimine dyes are commonly used in colour photography and in the production of pencils, as well as for dyeing paper and fur. In addition, they are used as chemical indicators [35].

**Figure 4:**
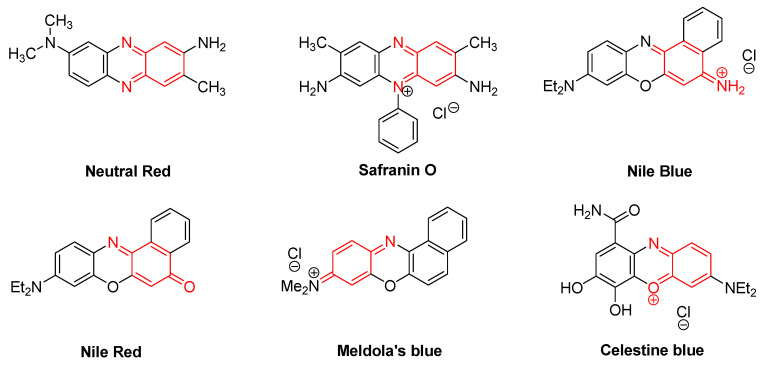
Some of the common quinoneimine dyes.

Some commonly used and important quinoneimine dyes are neutral red, safranin O, Nile blue, Nile red, Meldola’s blue, gallocyanin, gallamine blue, celestine blue B, and the methylene blue homologues (Figure 4). 

In microbiology, neutral red is used in the MacConkey agar to differentiate bacteria for lactose fermentation [36]. It also acts as a pH indicator, changing from red to yellow between pH 6.8 and 8.0.

Safranin is the classic counterstain in both Gram stains and endospore stains. It is also used as redox indicator in analytical chemistry.

Nile blue and Nile red are fluorescent dyes [37]. They have reasonably high fluorescence quantum yields in nonpolar solvents and they fluoresce at reasonably long wavelengths [38].

Meldola’s blue dye is used mainly as a pigment in textiles, paper, and paints. It has also been used in electrochemical experiments involving DNA, wherein the dye mediates electron transport [39].

Celestine blue dye is used with iron-aluminium complexes as a substitute for haematoxylin in H–E (haematoxylin–eosin) staining because of its resistance to low-pH solutions. It has been used as a new electroactive indicator in DNA biosensors and is also applicable to HOCl detection in living cells and to assaying the chlorinating activity of myeloperoxidase [40].

### 1.3. Biological Activity of N-Phenylquinoneimine Scaffolds

Quinoneimines are key abiotic and biological components that intercalate with DNA [11]. Quinoneimines and diimines are of interest in chemistry, and the former moieties have been proposed as intermediates in a number of biological processes [41]. Their diverse biological activities and synthetic applications have attracted the synthetic community to synthesise these important alkaloids [42,43,44,45]. Imai S. et al. [10] have reported exfoliazone (Figure 2), a phenoxazine antibiotic showing antifungal activity against *V. ceratosperma*. Pitucamycin and Chandrananimycin D have been found to exhibit antiproliferative activities against a number of cell lines and only a weak cytotoxicity [33].

Chandrananimycins A–C (Figure 2), isolated from *Actinomadura* sp. Isolate M048, have been reported to have high biological activity against *Staphylococus aureus*, *Bacillus subtilis*, and *Streptomyces viridochromogenes* [31]. They have also exhibited antialgal activity against the microalgae *Chlorella vulgaris*, *Chlorella sorokiniana*, and *Scenedesmus suspicatus* and antifungal activity against *Mucor miehei* and *Candida albicans*. Compounds such as Chandrananimycins A–C, containing the phenoxazine-3-one chromophore, are frequently encountered as metabolites of microorganisms. They are yellow-to-orange-coloured compounds and exhibit antibacterial [46], antifungal [10], phytotoxic [47], and anticancer activities. In addition, some are also known to show potent cell-growth-stimulating activity [9]. Due to their DNA intercalation, these complex phenoxazinone derivatives have shown pronounced antimicrobial [48], antitumor [49], and anticancer potency [50], with some of them also exhibiting anticoccidial activity [51]. Table 1 summarises the quinoneimine derivatives occurring as natural products and their biological activities.

### 1.4. Significance of Quinoneimine-Based Drugs

Exfoliazone (Figure 2) is an antibiotic that is active against *Valsa ceratosperma*, the causative fungus of the apple canker disease [10]. 

The actinomycins are a family of chromopeptide antitumor antibiotics isolated from various *Streptomyces* strains [50]. Actinomycins C_3_ and D have found clinical application as anticancer drugs, particularly in therapy for Wilm’s tumor [52] and soft tissue sarcomas [53] in children, and are still of interest in molecular biology [50]. 

**Figure 5:**
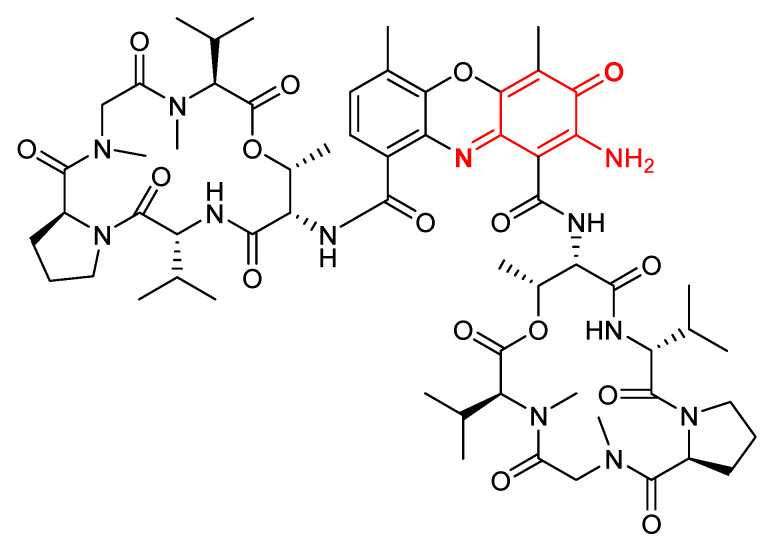
Structure of Actinomycin D.

Actinomycin D (Figure 5) has also been proposed as a therapeutic agent for AIDS, because of its potency as an inhibitor of HIV-1 minus-strand transfer [54]. It is a chemotherapy medication used to treat a number of types of cancer. This includes rhabdomyosarcoma, Ewing’s sarcoma, trophoblastic neoplasm, testicular cancer, and certain types of ovarian cancer [55].

Some chemicals and drugs have been reported to form reactive quinone and quinoneimine metabolites [56]. Quinoneimines are found as highly redox-active molecules and electrophiles, with both properties being crucial for their reactivity in biological systems. They are highly reactive organic chemicals and comprise a class of toxicological intermediates [57,58] that interact alone by generating reactive oxygen species (ROS) in biological systems to promote inflammatory reactions and reactive immune cells, oxidise DNA, and induce toxicity. They can be responsible for effects in vivo, including immunotoxicity, cytotoxicity, and carcinogenesis [57]. Quinoneimines reduce the oxygen to reactive oxygen species, acting as prooxidants, and, as electrophiles, they form covalent bonds with tissue nucleophiles.

**Figure 6:**
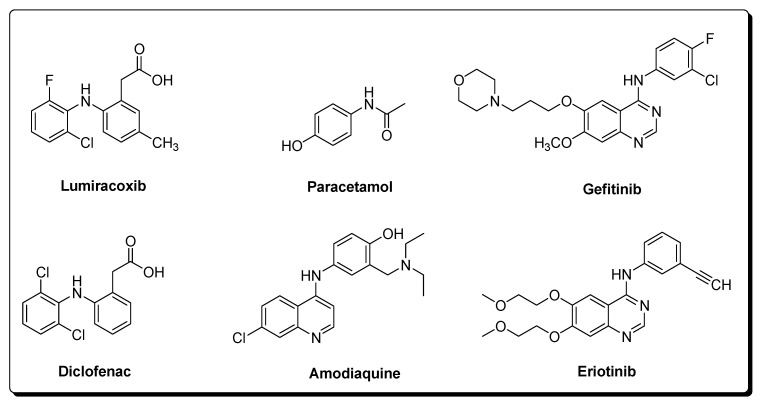
Some drugs that form quinoneimine reactive metabolites [56].

Important drug molecules that lead to the formation of quinoneimine reactive metabolites include Lumiracoxib (non-steroidal anti-inflammatory), Diclofenac (non-steroidal anti-inflammatory), Paracetamol (antipyretic), Amodiaquine (antimalarial), Gefitinib (kinase inhibitor), and Eriotinib (kinase inhibitor) (Figure 6).

**Scheme 1:**
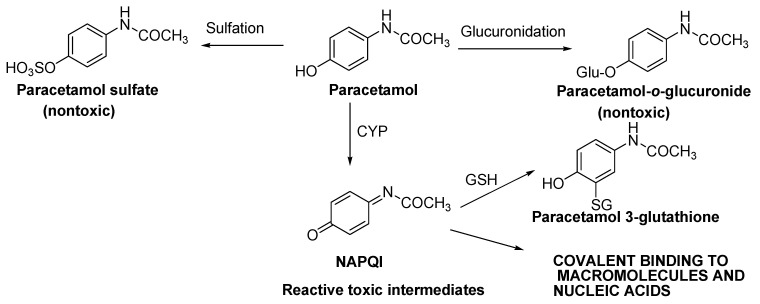
Paracetamol bioactivation to reactive species (quinoneimine) [56].

Paracetamol is widely used as an over-the-counter remedy to treat fever and pain. N-acetyl-p-aminophenol (APAP), the active ingredient in Paracetamol, is metabolised via 3 pathways: glucuronidation, sulfation, and glutathione conjugation. Glucuronidation and sulfation produce nontoxic metabolites for excretion. *N*-acetyl-p-benzoquinoneimine (NAPQI) is a toxic intermediate produced via cytochrome P450 2E1 (CYP 2E1; the main metabolising agent) and cytochrome P450 3A4 (CYP3A4) metabolism. NAPQI is then conjugated by glutathione (GSH) to form a nontoxic metabolite for excretion (Scheme 1) [56].

### 1.5. Synthesis of Quinoneimines

The common method of preparing *N*-phenylquinoneimines is through the oxidation of the corresponding hydroxydiphenylamine by using various oxidising agents (Table 2).

**Scheme 2:**
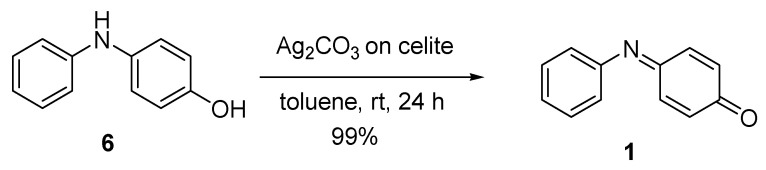
Preparation of *N*-phenylquinoneimine **1** [60].

Other oxidising agents used include iodoxybenzene and iodosylbenzene [68,69]. The use of Ag_2_CO_3_ on Celite for the oxidation of 4-hydroxydiphenylamine **6** is shown in Scheme 2. The Ag_2_CO_3_ adsorbed onto Celite, also known as Fétizon’s reagent, is a solid-supported oxidising agent (and so is easily removed after the reaction); it provides the iminoquinones in an excellent yield and is preferred to other methods [60,70]. The method uses two equivalents of Ag_2_CO_3_ and, at room temperature, 99% conversion to product **1** is observed in 24 h.

Most of these methods have disadvantages, such as the use of solvents and toxic reagents, a robust play environment, and low efficiency due to polymerisation, hydrolysis, and dimerisation. Electrochemical methods, on the other hand, are known as suitable, moderate, economical, fast, and easy methods that have been used for the synthesis of new quinoneimine derivatives [71].

## 2. Molecular Diversity from *N*-Phenylquinoneimine

Molecular diversity refers to the variety of different molecules that make up living organisms and their interactions with each other. This diversity is essential for life, as it enables organisms to carry out a range of biological functions, such as metabolism, growth, and reproduction. One aspect of molecular diversity that refers to the variety of chemical structures and properties of molecules is chemical diversity. Chemical diversity is important for drug discovery and development, as different molecules can have different effects on biological systems. Natural products, such as those derived from plants and microorganisms, are a rich source of chemical diversity and have been used for centuries in traditional medicine.

The best, but most difficult, strategy for chemical diversity is to make compounds in a way that anticipates problems at each step of the drug-discovery process in which organic synthesis is involved. The first such step involves finding a molecule that modulates a disease target or process; this requires thousands of structurally diverse compounds to be produced for screening. The next step is to optimise the biological properties of the compounds found during screening. This involves making analogues of the compounds, each containing slightly different structural modifications—ideally, every atom in the compound should be modified, without an overwhelming synthetic effort. The final step involves synthesising the optimal compound, either for use as a biochemical probe for research or as a drug in medicine, efficiently, at low cost and in large quantities [72].

**Scheme 3:**
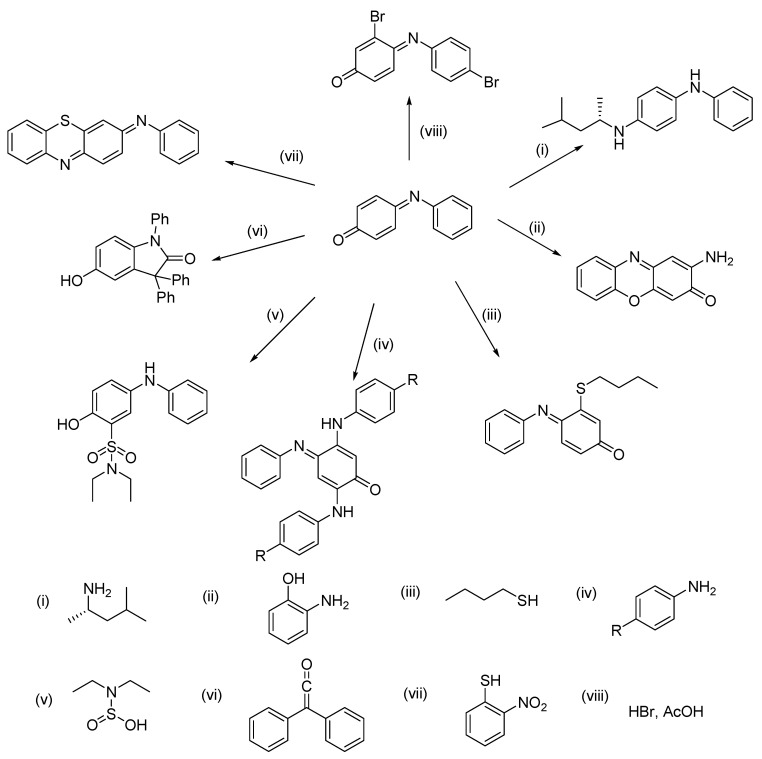
Reactions of *N*-Phenylquinoneimine leading to structural complexity [21].

Scheme 3 [21] shows some reactions of *N*-Phenylquinoneimine that can lead to structural complexity and molecular diversity.

### 2.1. Nucleophilic Addition Reactions of N-Phenylquinoneimine

#### 2.1.1. Direct Addition

**Scheme 4:**
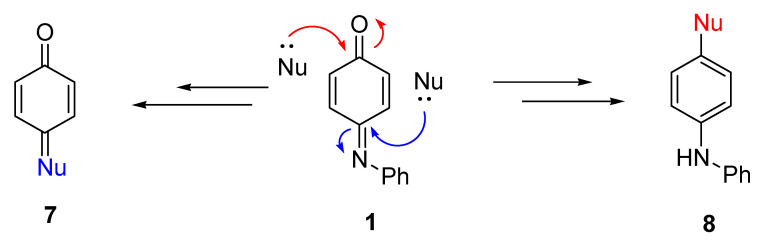
Nucleophilic attack via 1,2-addition to the ketone or the imine.

NPQ **1** can react with nucleophiles via 1,2-addition or 1,4-addition. NPQ has two unique sites susceptible to 1,2-addition: the ketone and the imine. If the nucleophile is an amine, for example, RNH_2_, then attack at the carbonyl carbon followed by reduction gives 1,4-diaminoarenes **8**, whilst attack at the imino carbon converts the *N*-phenyl group to a new group **7** (Scheme 4).

#### 2.1.2. Conjugate Addition

**Scheme 5:**
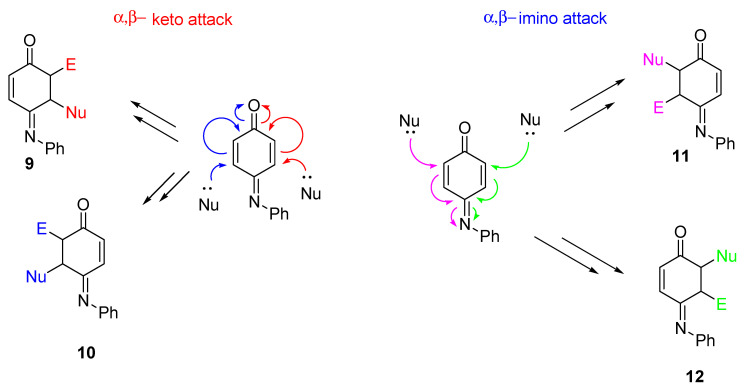
Nucleophilic attack via 1,4-conjugate addition.

There are two possible α,β-unsaturated systems in NPQ chemistry: α,β-keto and α,β-imino. All four β-carbons are unique and have different reactivities. Scheme 5 shows the products of the attack of a nucleophile at all four sites susceptible to 1,4-addition. By quenching the enolate with an electrophile other than H^+^, it may be possible to selectively add two substituents to the ring in a single reaction. The stereochemistry of the *N*-arylamine also introduces asymmetry. Attack on the α,β-ketone generates products **9** and **10**, whilst attack on the α,β-imine generates products **11** and **12** (Scheme 5).

### 2.2. Reaction with Nitrogen Nucleophiles

#### 2.2.1. Aliphatic Amines

**Scheme 6:**
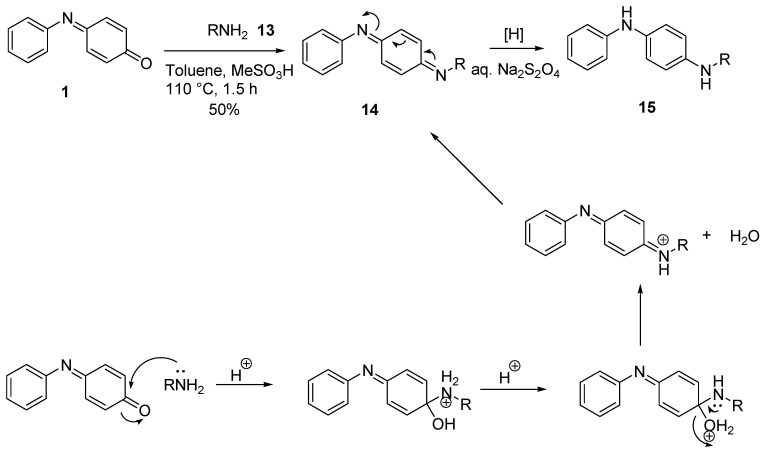
Addition of aliphatic amine to NPQ [21,66].

The addition of primary aliphatic amines **13** to NPQ **1** has been reported by Cottman [66,73] as a method of preparing *N*-substituted phenylenediamines. The reaction proceeds via a 1,2-addition to the carbonyl group, which, on reduction, gives 1,4-diaminoarenes (Scheme 6). The reaction between NPQ and primary amines may be in the presence of, or absence of, an acid catalyst. The *N*-phenyl-*N*′-diimines compound **14** was reduced to the *N*-substituted phenylenediamines **15** using aqueous sodium hydrosulfite. Representative catalysts for the hydrogenation reaction are platinum on carbon, palladium on carbon, and aqueous sodium hydrosulfite [66]. The amines that have been reported for the reaction includes methylamine, octylamine, 1,3-dimethylbutylamine, and cyclohexylamine. These *N*-substituted phenylenediamines have utility as anti-degradants in rubber, polymer stabilisers, dye intermediate, pharmaceuticals, and photographic chemicals [74,75].

#### 2.2.2. Aromatic Amines

**Scheme 7:**
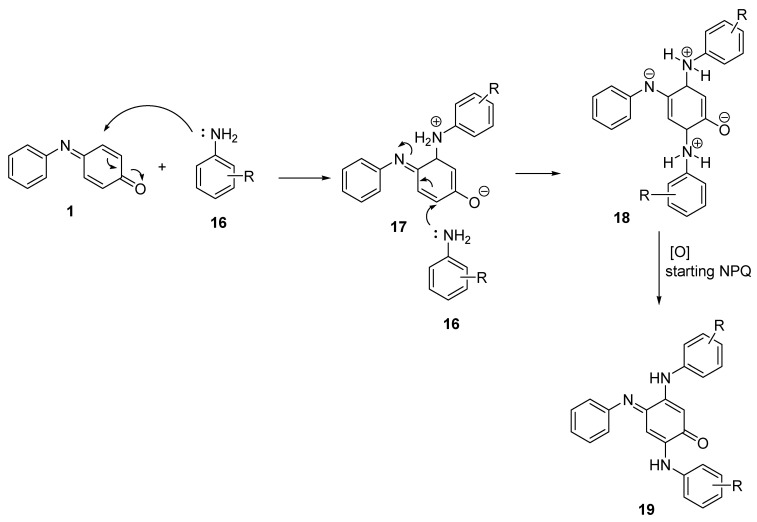
The reaction mechanism of the 1,4-conjugate addition of aromatic amines to the α,β-unsaturated ketone and α,β-unsaturated imine of NPQ [21,76].

The reactions of aromatic amines **16** with NPQ proceed through a 1,4-conjugate addition to the α,β-unsaturated ketone, yielding intermediate **17** (Scheme 7). This intermediate is then reacted with another equivalent of NPQ at the α,β-unsaturated imine site, resulting in intermediate **18**. Subsequent oxidation reactions lead to the formation of the desired diarylamino products **19**, as depicted in Scheme 7 [21]. The decrease in the oxidation potential of the quinoneimine, NPQ, is attributed to the introduction of electron-donor arylamino substituents. Consequently, the starting quinoneimine, NPQ, acts as an oxidising agent, as evidenced by the formation of p-hydroxydiphenylamine **19** [76]. 

**Scheme 8:**
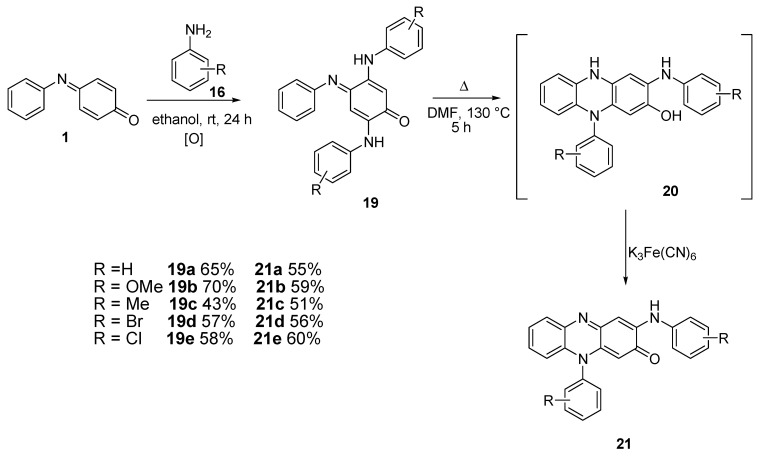
Reaction of aromatic amines with NPQ and subsequent oxidation to phenazinones [76].

The reaction has been reported by Tsoi E. V. et al. [76] Electron-withdrawing and electron-donating anilines have been used, giving good yields (Scheme 8). Aromatic amines react with NPQ, forming diarylamino products **19**, which, when heated under oxidative conditions (using potassium ferricyanide), are converted to products of intramolecular oxidative cyclisation [76], phenazinones **21** through 5,10-dihydrophenazine derivatives **20** (Scheme 8).

The cyclisation of substituted quinone imines and diazabutadiene derivatives of aminophenols afforded phenoxazine and benzoxazine derivatives, which were finally transformed into fused heterocyces [77].

### 2.3. Reaction with Sulfur Nucleophiles

NPQ reacts with sulfur nucleophiles, such as thiols [78], thiophenols [79], and sulfinates [80], via nucleophilic addition.

#### 2.3.1. Thiols

**Scheme 9:**
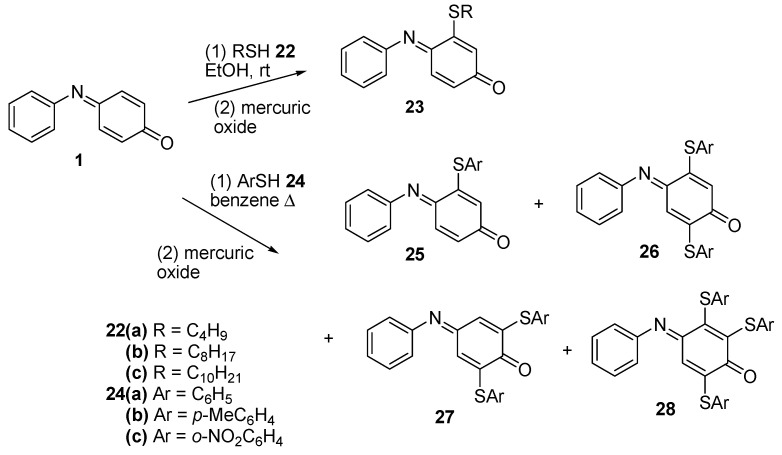
Reactions of thiols with NPQ [78].

The nucleophilic addition of alkane- and arenethiols to NPQ occurs at the α,β-unsaturated imine and the α,β-unsaturated ketone [78]. The reaction with alkane thiols **22** was conducted in ethanol at room temperature, giving the products of the hydroxydiphenylamines, which are then oxidised using mercuric oxide to the corresponding alkylthio- quinonimines **23**. The reaction with arenethiols **24**, however, was carried out in benzene and refluxed; the alternative addition products **25**, **26**, **27**, and **28** were observed (Scheme 9). The difference in these reactions is attributed to the solvent effect of the polar protic solvent (ethanol) versus a nonpolar solvent (benzene). The reaction of *N*-Phenyl-1,4-benzoquinonemono imine benzoanalogs with alkanethiols was found to give similar 1,4-addition products [81].

**Scheme 10:**
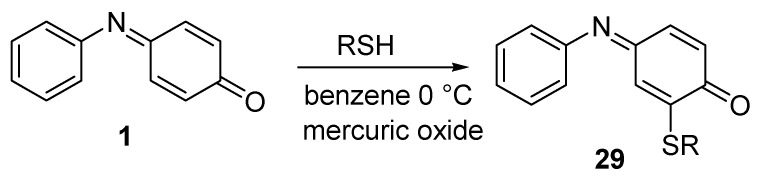
Alternative mode of addition of thiols to NPQ.

With a change in the reaction conditions (0 °C in benzene), the 6-monosubstituted derivatives **29** was formed (Scheme 10).

#### 2.3.2. Sulfinates

**Scheme 11:**
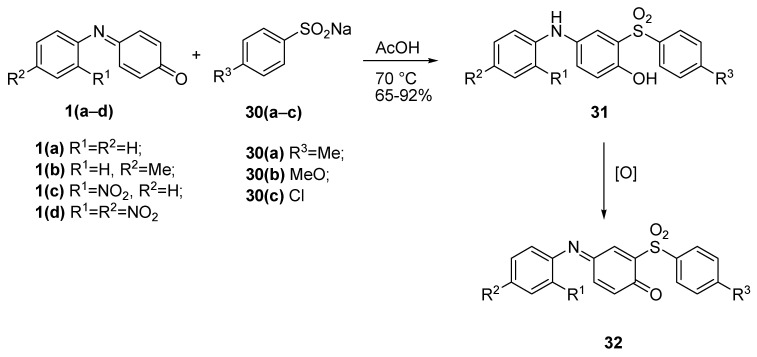
Addition of sodium arenesulfinates to NPQ derivatives [80].

The reaction of NPQ derivatives with sodium arenesulfinates **30** has been reported by Konovalova et al. [80]. The reactions were carried out in acetic acid at 70 °C using two equivalents of the nucleophile; however, the reactions afforded, exclusively, the product of single 1,4-addition to the α,β-unsaturated imine system **31**. This was then oxidised with lead (IV) acetate to the corresponding 1,4-benzoquinone imine derivatives **32** (Scheme 11). This method provides an alternative route to making this type of sulfone. 

**Figure 7:**
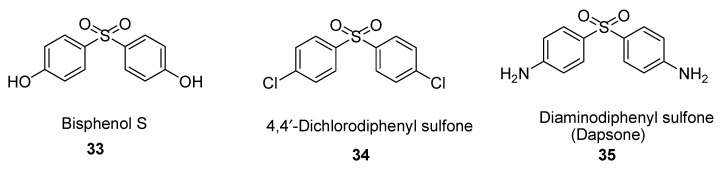
Some useful sulfone-containing compounds.

Some polymers containing sulfone groups are useful engineering plastics, as they exhibit high strength and resistance to oxidation, corrosion, high temperatures, and creep under stress; for example, some are valuable as replacements for copper in domestic hot-water plumbing [82]. Precursors to such polymers are the sulfones bisphenol S **33** and 4,4′-dichlorodiphenyl sulfone **34** (Figure 7). Examples of sulfones in pharmacology include dapsone **35**, a drug formerly used as an antibiotic to treat leprosy. Several of its derivatives, such as promin, have similarly been studied or applied in medicine [83].

### 2.4. Reaction with Halogens

**Scheme 12:**
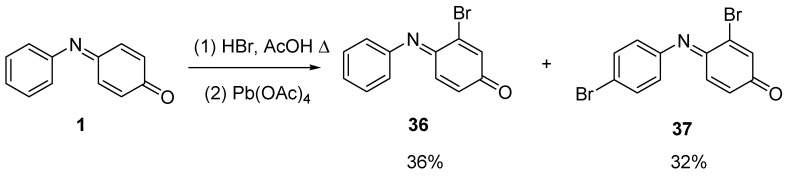
Reaction of HBr with NPQ [84].

Initial studies on the reaction of hydrobromic acid with NPQ have been reported by Burmistrov et al. [84]. It was reported that the nucleophilic addition of bromide was to the α,β-unsaturated ketone, giving compound **36** (Scheme 12). Aqueous hydrobromic acid was added to NPQ in acetic acid at room temperature, and organic extracts from the reaction mixture were treated with lead (IV) acetate to convert the phenol intermediate to the quinoneimine system. Bromination of the phenyl ring at the para position was also found to occur as a competing process, giving the di-substituted compound **37**. The addition of resorcinol to the reaction of NPQ with hydrobromic acid clearly changed the composition of the reaction mixture, eliminating the bromination of the phenyl group and giving the mono-substituted product **36** only.

**Scheme 13:**
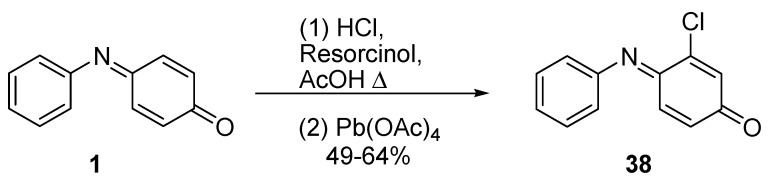
Addition of hydrochloric acid to NPQ [85].

The reaction of hydrochloric acid with NPQ was conducted in the same manner and reportedly gave the mono-substituted compound **38**. However, the chlorination of the phenyl group was not observed (Scheme 13) [85].

### 2.5. Reaction with Carbon Nucleophiles

#### 2.5.1. Enolates

**Scheme 14:**
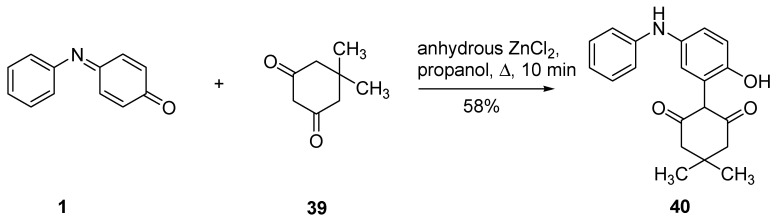
Reaction of NPQ with dimedone [86].

The reaction of NPQ with dimedone **39** has been reported by Novikov V. P. et al. [86]. The condensation reaction was carried out by boiling the quinoneimine, dimedone, and anhydrous zinc chloride in propanol for 10 min. The reaction mixture was worked up with water after cooling for 12 h at room temperature. The resulting product was filtered and washed, giving a yield of 58% of compound **40** (Scheme 14).

#### 2.5.2. Organometallic Reagents

**Scheme 15:**
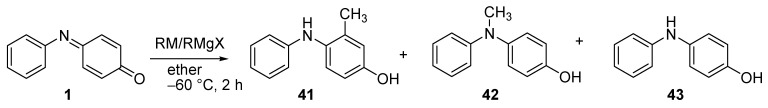
The reaction of NPQ with organometallic reagents [87].

The reaction of NPQ with organometallic reagents gave the products of 1,4-addition **41,** 1,6-addition **42**, and reduction **43** (Scheme 15) [87].

The course of the reactions with the organometallic reagents used is shown in Table 3 below.

## 3. Oxidation of *N*-Phenylquinoneimine

**Scheme 16:**
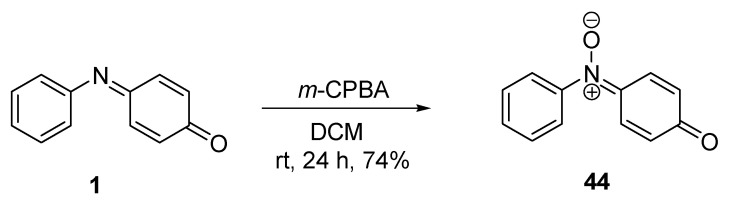
Oxidation of NPQ.

The oxidation of NPQ using meta-Chloroperoxybenzoic acid in DCM at room temperature yielded the *N*-phenylquinoneimine *N*-Oxide **44** (Scheme 16) [88].

The presence of an electron-withdrawing group should cause a change in the chemical shifts in the ^1^H NMR spectrum of NPQ *N*-oxide when compared to NPQ. The largest differences will be for protons closest to the nitrogen, as expected when considering their proximity to the *N*-oxide. A relatively smaller downshift in the peaks for the other protons, which are farther away from the oxygen of the *N*-oxide, should, therefore, be expected.

### 3.1. Cycloaddition Reactions

**Scheme 17:**
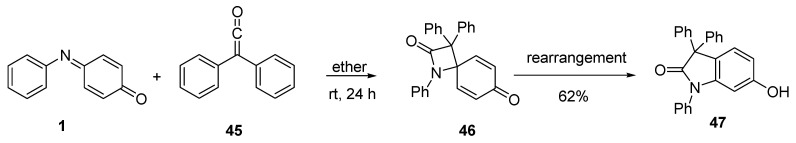
Cycloaddition reaction of NPQ with diphenylketene.

The addition of diphenylketene **45** to NPQ in ether, as reported by Bird C. W. [89], resulted in the formation of a cycloaddition product, β-lactam **46**, which rearranged to give the oxindole **47** (Scheme 17). The oxindole can be readily converted into the methyl ether via dimethyl sulphate and methanolic sodium hydroxide.

### 3.2. Coupling Reactions

Some palladium-catalysed coupling reactions of NPQ have been investigated using the monosubstituted bromo-NPQ **36**. [21] The Suzuki, Stille, Kumada, and Buchwald–Hartwig reactions of NPQ are discussed below; others include carbonylation [90] and redox-neutral C-N coupling reactions [91].

#### 3.2.1. Suzuki Coupling

**Scheme 18:**
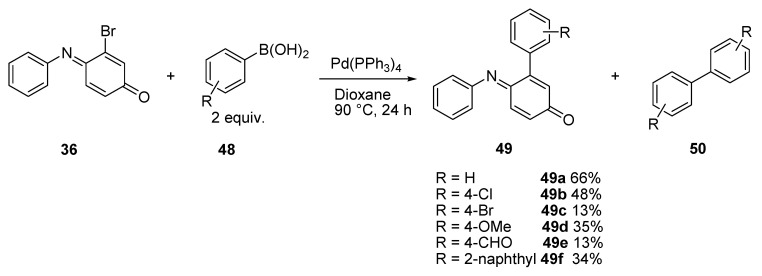
Suzuki reaction of compound **36** with arylboronic acids.

The reaction of 3-bromo NPQ **36** with a range of arylboronic acids gave the coupled products **49** in average-to-good yields, with the biaryl products **50** obtained as by-products resulting from the homocoupling of the arylboronic acid (Scheme 18) [21]. A much lower yield was observed when electron-deficient boronic acids were used, and the equivalent of arylboronic acid used was found to be insignificant to the yield of the coupled product.

#### 3.2.2. Stille Coupling

**Scheme 19:**
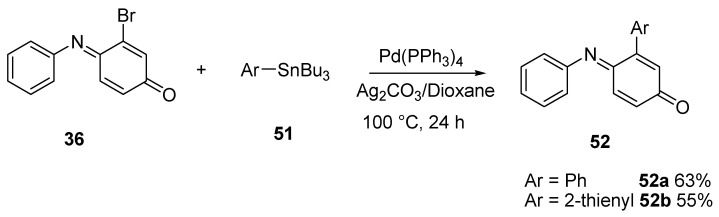
Stille reaction of compound **36** with arylstannanes [21,92].

The reaction of 3-bromo NPQ **36** with tributyl(phenyl)stannane **51a** was carried out using one equivalent of the arylstannane in THF, with LiCl as the base, and heated at 100 °C for 24 h [92]; both the coupled product and biaryl product were obtained in low yields. The reaction was repeated with the base changed to Ag_2_CO_3_ and the solvent to dioxane, and an increase in yield was observed for the coupled product **52**, while the biaryl product was not formed (Scheme 19). This demonstrates that the solvent/base combination is critical in the optimisation of the palladium-catalysed Stille reaction. The reaction with tributyl(thiophen-2-yl)stannane **51b** was, therefore, only carried out using Ag_2_CO_3_ in dioxane, and the coupled product was obtained at a good yield, while the biaryl product was not formed. This selectivity could also result from the use of one equivalent of the arylstannane, since the reaction of a Pd(II) precatalyst with two equivalents of the arylstannane mostly accounts for the homocoupling side-products [93].

#### 3.2.3. Kumada Coupling

**Scheme 20:**
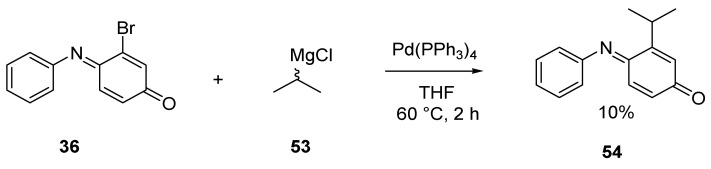
Reaction of 3-bromo NPQ with isopropylmagnesium chloride [21,91].

The reaction of 3-bromo NPQ **36** with isopropylmagnesium chloride **53** was conducted in THF at 60 °C for 2 h (Scheme 20) [94]. The crude reaction mixture was purified via chromatography (SiO_2_ 1:4 ethylacetate–petrol) to give the coupled product **54** as a red oil. The Kumada coupling, although only giving a low yield of the product, is important and different from the other coupling reactions investigated, as it allows the introduction of alkyl as well as aryl groups.

The lack of reactivity and low yields observed in some of the palladium-catalysed coupling reactions considered may result from the oxidation of Pd(0) to Pd(II) by the bromo NPQ in the oxidative addition step of the catalytic cycle, since it also acts as an oxidising agent.

#### 3.2.4. Buchwald–Hartwig Coupling

**Scheme 21:**
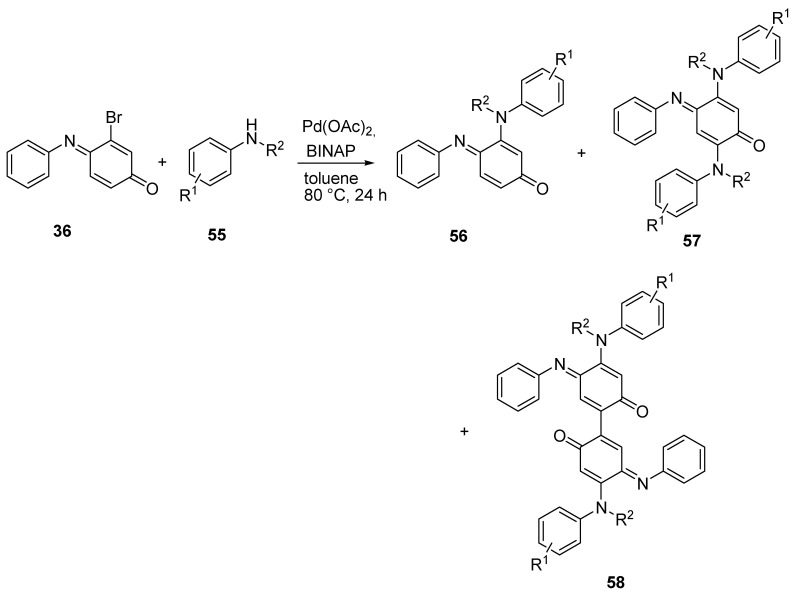
Reaction of 3-bromoNPQ with aniline derivatives [21].

The Buchwald–Hartwig amination reaction was investigated by coupling 3-bromo NPQ with arylamines as a means of forming new C–N bonds (Scheme 21) [21].

The reaction, which may also occur via addition to the α,β-unsaturated ketone, gives the expected coupled product **56** in good yields, as shown in Table 4. The anilines with electron-deficient groups (entries 3 and 4) were observed to give lower yields. The product of diaddition to the α,β-unsaturated ketone and α,β-unsaturated imine, **57** was also observed. A product of dimerisation was observed with entries 5 and 6, giving compound **58** as a by-product resulting from oxidative coupling between two molecules.

The formation of the dimerised product **58** is not surprising, as it has been reported that unexpected observations, new transformations, or unusual side reactions often occur in Pd-catalysed reactions, and these stem from poor catalytic turnover, unusual reactivity or selectivity, or the presence of unwanted side-products [93].

## 4. Conclusions

Overall, *N*-phenylquinoneimine-based compounds have emerged as a powerful probe for scientists in drug discovery and medicinal chemistry. Significant advances have been evidenced in their natural product chemistry, copolymerisation [95], and coupling reactions. This novel class of quinoneimine derivatives shows promising antibacterial, antimicrobial, anticancer, anti-inflammatory, antioxidant, and other therapeutic activities that position them as versatile potential drug candidates for interventions in various disease areas and for use as building blocks to access new synthetic molecules.

## Figures and Tables

**Table 1:** Naturally occurring quinoneimines and their biological activities.

Quineimine Derivatives as Natural Products	Biological Activities (References)
Exfoliazone	Antibiotic, antifungal, antitumor, growth-promoting activities [9,10]
Questiomycin A, *N*-acetylquestiomycin A, and Acetylmichigazone	Growth stimulatory and inhibitory effects [9]
Venezuelines A–G	Cytotoxic and antitumor activities [30]
Chandrananimycins A–D	Antibacterial, antifungal, antialgal, phytotoxic, and anticancer activities [31,33]
Actinomycins	Antibacterial, antitumor, and anticancer activities [52,53,54,55]
Pitucamycin	Antiproliferative and cytotoxic activities [33]
Grixazone B	Antimicrobial [33]
Benzerramycin A–C	Antiproliferative [32]
Cinnabarin and Cinnabarinic acid	Antibacterial, antimicrobial [34]

**Table 2:** Various oxidising agents used in the preparation of quinoneimines.

Substrate	Oxidising Agent	Solvent	Temp (°C)	Time (h)	Yield (%)	Ref.
**6**	HgO	Benzene	Reflux	1	78	[59]
**6**	Ag_2_CO_3_ on Celite	Toluene	rt	30 min	99	[60]
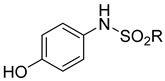	Pb(OAc)_4_	Acetic acid	rt	1	58	[61]
**6**	Hypochlorite	Heptane	rt	1	99	[62]
**6**	Hydrogen peroxide	Toluene	35	25 min	99	[63]
**6**	Activated carbon catalyst	Methanol	50	1	90	[64]
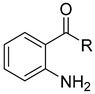	2-iodoxybenzoic acid	Dimethyl sulfoxide	rt	35 min	84–98	[20]
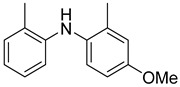	Dess–Martin periodinane	Dichloromethane	rt	8	56	[65]
**6**	K_2_Cr_2_O_7_	Acetone	40	45 min	97	[66]
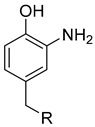	K_3_[Fe(CN)_6_]	Phosphate buffer	rt	30 min	n.r.	[67]

**Table 3:** Reaction of NPQ with organometallic compounds at −60 °C.

Reagent	Solvent	41 (%)	42 (%)	43 (%)
CH_3_Li	THF ^a^	9.8	9.3	9.8
CH_3_Li	Ether	13.6	14.1	13.9
CH_3_MgI	Ether	15.6	14.0	14.8
CH_3_CuLiI	Ether	0	30.5	28.7
CH_3_CuLiI	THF ^a^	0	28.1	57.7
CH_3_Li + CuCl ^b^	THF ^a^	0	44.0	7.8
CH_3_MgI + CuI ^c^	Ether	23.4	17.8	18.4

^a^ Tetrahydrofuran; ^b^ a solution of methyllithium was added to the mixture of the substrate and CuCl (1:2) at 25 °C while stirring; ^c^ 5 mol% of CuI (referred to the reagent) was added to the solution of the Grignard reagent at −15 °C, and the solution was stirred for 30 min at this temperature, then cooled to −60 °C, and the reaction was effected.

**Table 4:** Reaction of 3-bromo NPQ with aniline derivatives.

Entry	Aniline 55	R^1^	R^2^	56 (%)	57 (%)	58 (%)
**1**	**a**	H	H	79	13	nd
**2**	**b**	4-OMe	H	66	17	nd
**3**	**c**	4-Cl	H	29	15	nd
**4**	**d**	4-CF_3_	H	29	3	nd
**5**	**e**	2-Me	H	72	t	5
**6**	**f**	H	Me	59	8	3

nd = not detected, t = trace amount.

## Data Availability

The data presented in this study are available in article.
